# A separable neural code in monkey IT enables perfect CAPTCHA decoding

**DOI:** 10.1152/jn.00160.2021

**Published:** 2022-02-23

**Authors:** Harish Katti, S. P. Arun

**Affiliations:** Centre for Neuroscience, Indian Institute of Science, Bangalore, India

**Keywords:** decoding, neural mechanisms, object recognition, reading

## Abstract

Reading distorted letters is easy for us but so challenging for the machine vision that it is used on websites as CAPTCHA (Completely Automated Public Turing Test to tell Computers and Humans Apart). How does our brain solve this problem? One solution is to have neurons selective for letter combinations but invariant to distortions. Another is for neurons to encode letter distortions and longer strings to enable separable decoding. Here, we provide evidence for the latter possibility using neural recordings in the monkey inferior temporal (IT) cortex. Neural responses to distorted strings were explained better as a product (but not sum) of shape and distortion tuning, whereas by contrast, responses to letter combinations were explained better as a sum (but not product) of letters. These two rules were sufficient for perfect CAPTCHA decoding and were also emergent in neural networks trained for word recognition. Thus, a separable neural code enables efficient letter recognition.

**NEW & NOTEWORTHY** Many websites ask us to recognize distorted letters to deny access to malicious computer programs. Why is this task easy for our brains but hard for the computers? Here, we show that, in the monkey inferior temporal cortex, an area critical for recognition, single neurons encode distorted letter strings according to highly systematic rules that enable perfect distorted letter decoding. Remarkably, the same rules were present in neural networks trained for text recognition.

## Graphical abstract

**Figure:**
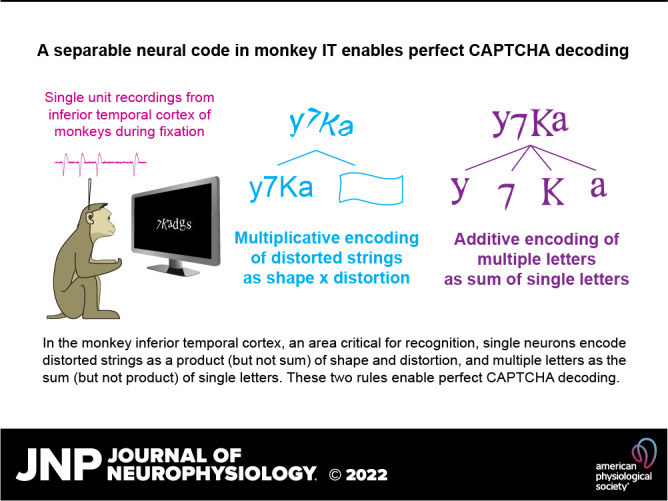

## INTRODUCTION

**Figure 1.**
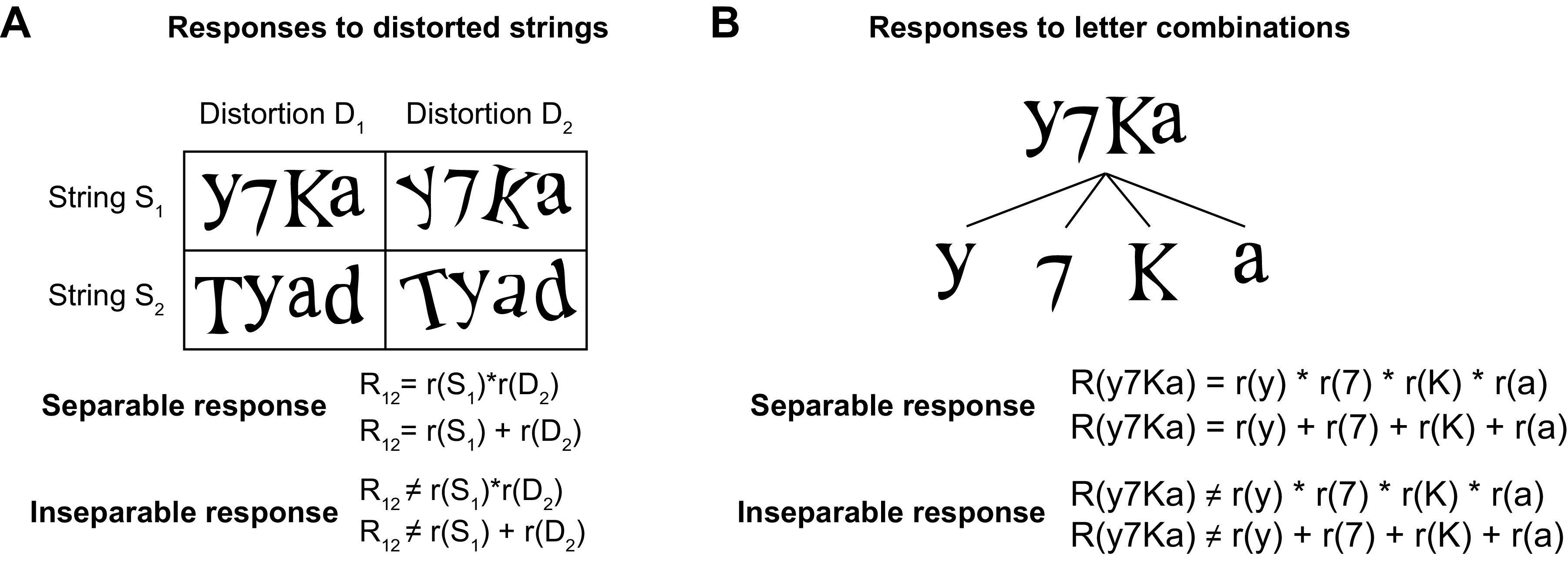
Separability of distortions and letter combinations. *A*: we define the neural response to distorted letters or strings as separable if the response to a letter or string at a particular distortion can be understood as either a sum or product of string tuning and distortion tuning, and inseparable otherwise. Note that the underlying distortion tuning cannot be measured directly but can be estimated using a suitable model. *B*: we define the neural response to letter combinations as separable if the response to a particular letter combination (e.g., y7Ka) can be understood as a sum or product of the individual letter responses (e.g., y, 7, K, and a), and inseparable otherwise. Inseparable responses could occur for instance if a neuron responds selectively to a specific combination of letters but does not respond to the constituent letters. Note that responses to individual letters could either be measured directly or indirectly estimated by measuring responses to many letter strings containing the same set of letters.

Reading relies upon our ability to recognize letters and words despite variations in appearance. These include variations due to size, location, or viewpoint but also arising from writing styles, font, and distortions of the writing surface. Yet, we do solve this task so much better than computers that it is exploited on websites as CAPTCHA (Completely Automated Public Turing Test to tell Computers and Humans Apart) (1). How does the brain solve this problem? A widely held view is that neurons in higher visual areas are invariant to letter distortions and selective for specific combinations of letters (2, 3) or features. In this scenario, neural responses to letter combinations cannot be understood using single letters because of the presence of emergent features (Fig. 1). This view is supported by observations that neurons in the monkey inferior temporal (IT) cortex, an area critical for recognition (4, 5), contain neurons invariant to size, location, and viewpoint (6–8) but selective for feature combinations (9–12). This selectivity for feature combinations increases with discrimination learning (2, 12).

An alternate possibility is that neurons may encode letters and their distortions as well as longer strings of letters in a separable fashion (Fig. 1). By separable encoding we mean that responses to letter distortions or combinations can be understood in terms of their constituent letter or distortion selectivity. Evidence for this account comes from recent studies showing that IT neurons separably encode object identity and invariant attributes (13) and respond to objects as a sum of their parts (14–16). This separability explains many aspects of orthographic processing (17) and increases with reading expertise in humans (18). This separability is also consistent with the finding that IT neurons preserve their shape/object preference across identity preserving transformations (6, 9, 19, 20), but the specific functional form of this separability has not been investigated extensively.

These possibilities have not been investigated in IT neurons by recording their responses to distorted letter strings, which are unlike natural objects in that their constituent units (letters) are spatially separated and can undergo not only identity-preserving transformations, such as changes in size and position, but also a wide variety of stylistic distortions. Thus, it is not clear if the pre-existing neural machinery in naive monkey IT itself suffices to solve CAPTCHAs.

We investigated this issue by recording neural responses in IT cortex of monkeys trained to fixate distorted letter strings but with no explicit training to discriminate or recognize these stimuli. We found that IT neurons encode distorted letter strings according to two systematic rules: letter distortions were encoded as a product of letter and distortion tuning; and letter combinations were encoded as a sum of single letters. To confirm that these rules are indeed sufficient to solve CAPTCHAs, we instantiated these rules in artificial neurons and asked whether these neural responses can be used to decode CAPTCHAs. Finally, we show that these rules naturally emerge in later layers of deep neural networks trained for letter and word recognition.

## METHODS

All experiments were performed in accordance with a protocol approved by the Institutional Animal Ethics Committee of the Indian Institute of Science and by the Committee for the Purpose of Control and Supervision of Experiments on Animals, Government of India. Most experimental procedures were common to previously reported studies in our laboratory (8, 13) and are only briefly summarized immediately below.

### Neurophysiology

We recorded from the left IT cortex of two macaque monkeys (*Macaca radiata*, Ka and Sa, both male, aged 7 yr) using standard neurophysiological procedures (8). Recording sites were located in the anterior ventral portion of IT. Extracellular wideband signals were recorded at 40 KHz using 24-channel laminar electrodes (Uprobes with 100-µm intercontact spacing, Plexon Inc.). We manually sorted these signals offline into distinct, well-isolated clusters using spike-sorting software (OfflineSorter, Plexon Inc.). Isolated unit waveforms, for example neurons are shown in Supplemental Fig. S5 (all Supplemental material is available at https://doi.org/10.17605/OSF.IO/2SGEU). Neurons were selected for further analysis if they had reliable responses to at least one of the stimuli during the image presentation period (0–200 ms) based on visual inspection of peristimulus time histograms (PSTH) constructed for each stimulus. We selected 139 visually responsive units in this manner for further analysis, all with well-isolated unit waveforms. Of these, only two units were from a single channel with distinct waveform shape and amplitudes, and all others were from distinct, separate channels. Post hoc analyses of the three-dimensional (3-D) recording locations (AP/ML/depth) revealed that the recording sites were spread across ∼4 mm along the anterior-posterior axis and 5 mm in the medial-lateral axis of the recording grid.

### Stimuli

We created a set of 432 unique stimuli. We selected eight letters which included lower- and upper-case letters and numbers (“a,” “d,” “g,” “s,” “T,” “y,” “7,” and “K”). Each single letter was rendered at six retinal locations 0.8° apart, three adjacent locations being ipsilateral, and three contralateral. Presenting each of the unique letter shapes at six unique spatial locations resulting in a total of 48 possible single letter placements. These locations were chosen to match the letter locations in the longer *n*-gram strings described in the next section. Letters were rendered in the Times New Roman font and subtended 0.8° visually on an average.

These letters were also used to create 10 bigrams, 10 trigrams all the way to 10 6-grams (Supplemental Fig. S1), resulting in a total of 50 unique *n*-grams. Thus, there were a total of 98 undistorted single letters and *n*-grams.

Next, we applied three different distortions drawn from commercial captcha algorithms to each of these 98 letter or *n*-gram placements. In the first, we applied a local fish-eye lens distortion that expands and translates pixels in *x* and *y* directions (https://in.mathworks.com/matlabcentral/fileexchange/22573-pinch-and-spherize-filter). We applied a second distortion over and above the first distortion which involved randomly rotations (uniformly chosen from −15° to 15°) of every character in the string. The third distortion was added over and above the second distortion and affected the global shape of the string. It involved bending the entire *n*-gram about one of two different spline curves. All distortions were implemented using custom scripts written in MATLAB. Since each stimulus was presented in its undistorted form and three distortions, this resulted in a total of 98 × 4 = 392 stimuli.

In addition to these distortions, we also included hard CAPTCHA variants used by Google, Yahoo, and other web services (Fig. 8, Supplemental Fig. S1*B*). We selected a subset of eight 6-gram strings (“7Kadgs,” “KadgsT,” “Ty7Kad,” “Tyadgs,” “gsTy7K,” “sTy7Ka,” “y7Kadg,” and “yadgsT”) which were distorted in four additional ways: *1*) rotating the letters in place so that they touched each other; *2*) retaining only the letter outlines; *3*) adding a fish-eye transformation, rotating letters in place, and adding a background line grid; and *4*) adding a fish-eye transformation, rotating letters in place (uniformly at random between 15° and −15°), and adding a wavy stroke cutting through the letters. These distortions can be seen in Fig. 8. These distortions are known to be challenging for computer vision algorithms and are used for human-vs-robot authentication tests (1) in commercial web services from Yahoo (touching solid letter and letter outline hard CAPTCHAs) and Google (hard CAPTCHAs with strike-through and background grid).

### Behavioral Task

Each animal was trained to fixate a series of eight images presented at fixation in return for a juice reward. Each image was shown for 200 ms with an interstimulus interval of 200 ms. Thus, each trial lasted 3.2 s. Trials contained either single letters or *n*-grams, randomly ordered with the constraint that the same stimulus should not repeat in a given trial. The 432 stimuli were organized into 54 blocks of 8 stimuli each, and we generated four unique sets of these 54 trials using pseudorandom assignment of letter or *n*-gram stimuli. During recordings, a set of 54 trials were completed (corresponding to one repetition of the entire stimulus set) before starting another set of 54 trials. *N*-grams with even number of letters (2, 4, and 6-grams) were centered on fixation whereas those with odd number of letters (3- and 5-grams) were positioned such that the contralateral side had one extra letter. At least four repetitions were obtained corresponding to each stimulus image, for every visually responsive neuron. More repetitions per stimulus, although desirable, were not feasible given the many constraints of extracellular recording duration.

### Calculation of Response Reliability

We calculated a measure of response reliability for each neuron, which represents an upper bound on model performance. This was simply the split-half correlation, i.e., the correlation between the average firing rate across all stimuli under consideration across the two odd-numbered and two even-numbered repetitions separately.

### Model Fits for Letter × Location × Distortion Encoding

**Figure 2.**
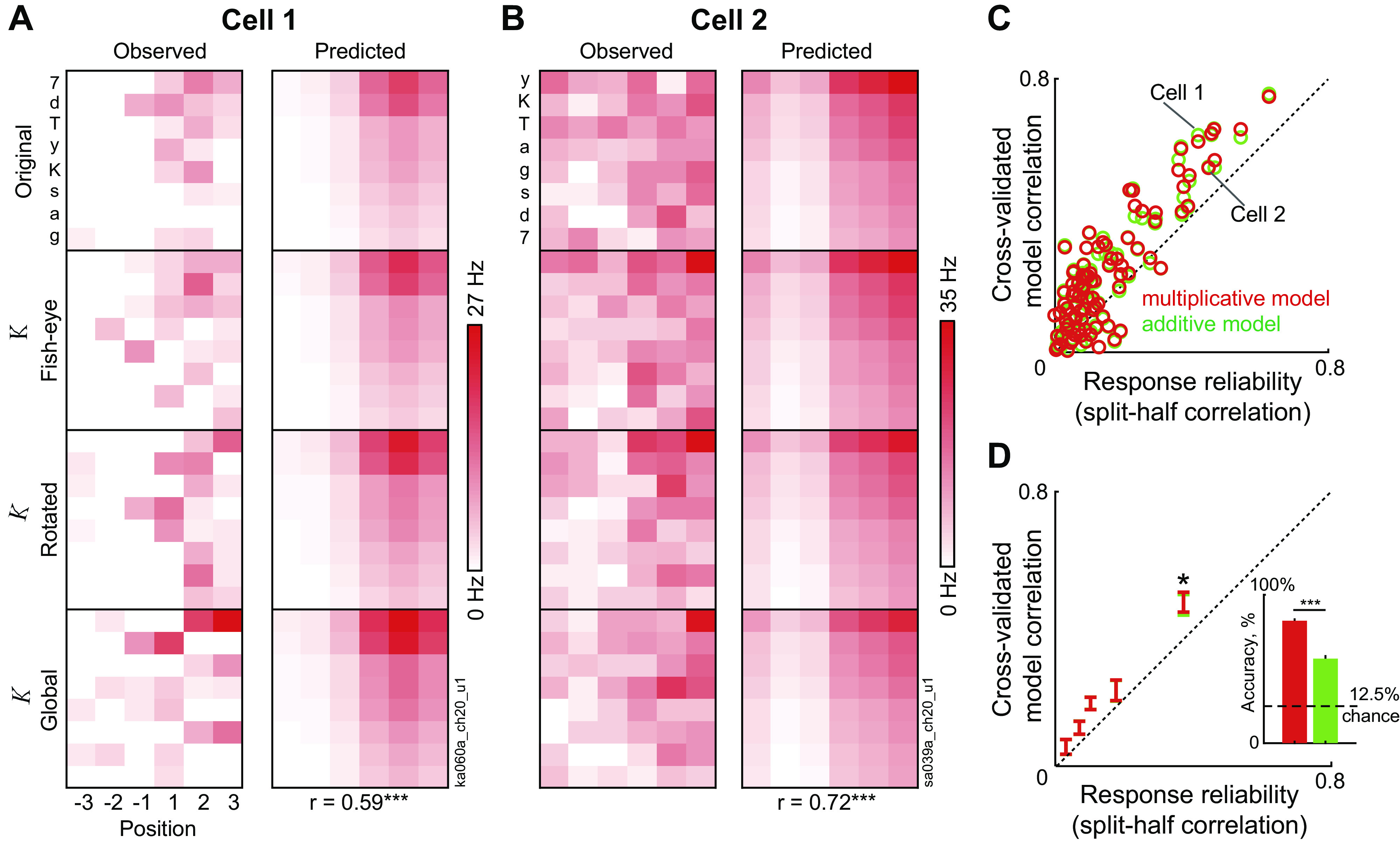
Inferior temporal (IT) neurons show separable encoding of distorted letters. *A*, *left*: colormap showing the firing rate of an example IT neuron (averaged across all trials) to single characters (arranged along the *y*-axis, from best to worst) at each retinal location (arranged along the *x*-axis from ipsilateral to contralateral). Each character was shown at four distortions (undistorted, fish-eye, rotated, and global). Darker colors indicate higher firing rate. *Right*: colormap depicting the predictions of the multiplicative model trained on the observed response (correlation with observed response: *r* = 0.59, *P* < 0.0005, indicated below). In the multiplicative (additive) model, responses to distorted letters at various positions are predicted as a product (sum) of shape, location, and distortion tuning. *B*: same as *A* but for another example IT neuron. *C*: cross-validated model correlation for the multiplicative (*red*) and additive (*green*) models plotted against response reliability (i.e. split-half correlation) across neurons with positive reliability. *D*: progression of model correlation with response reliability for neurons at each 20th percentile of response variability for the multiplicative (*red*) and additive (*green*) models. Error bars represent means ± SE in each bin. Asterisks above the last bar indicate a significant difference between the multiplicative and additive model predictions for the most reliable neurons (*P < 0.05, sign-rank test across 19 neurons). *Inset:* to investigate whether the slight advantage for multiplicative encoding of shape, position, and distortion at the single cell level has any consequence for letter decoding, we trained linear classifiers to decode letter identity using the multiplicative and additive model predictions separately (see methods). Decoding accuracy is shown for the multiplicative population (*red*) and additive population (*green*). Error bars represent standard deviation of accuracy. Asterisks represent statistical significance calculated as the proportion of the 10,000 cross-validated splits in which the multiplicative population accuracy was lower than the additive population (***P < 0.0001).

We tested each neuron with eight letters presented at six retinal locations and four distortions. We used the firing rate of each neuron (calculated in a 50- to 200-ms window after stimulus onset) to these 192 stimuli to fit two models. To do so, we first calculated the average shape tuning for the neuron by averaging its response (on odd-numbered repetitions) to every shape across retinal locations and distortions. We proceeded likewise to calculate the average retinal location tuning and average distortion tuning. For the additive model, the response to shape *i*, location *j*, and distortion *k* is given by *R*(*s_i_*, *p_j_*, *d_k_*) = *S_i_* + *P_j_* + *D_k_*, where *S_i_* is the average response to shape *i* (after averaging across locations and distortions), *P_j_* is the average response for location *i* and *D_k_* is the average response to distortion *k*. Likewise, for the multiplicative model, the response is given by *R*(*s_i_*, *p_j_*, *d_k_*) = *S_i_* × *P_j_* × *D_k_*. Both model predictions were scaled and shifted to be in the same range as the observed response. To evaluate the model fit, we calculated the correlation between the model predictions trained on odd-numbered repetitions and the firing rate of the neuron on even-numbered repetitions, and vice-versa. We averaged these two model correlations to obtain a cross-validated model correlation. For Fig. 2, *C* and *D*, we excluded 37 neurons with high response variability that caused them to have a negative split-half correlation. Our results remained qualitatively similar upon varying these inclusion criteria.

### Letter Decoding from Distorted Letters Using Multiplicative and Additive Populations

We fit additive and multiplicative models as described above for each neuron using its average firing rate (across all trials), and created two 139 × 192 response matrices (139 neurons × 192 letter stimuli) for the additive and multiplicative population. We also created a 192 × 1 vector representing the letter that was shown in each case. We then trained linear classifiers for eight-way letter classification on the additive and multiplicative populations separately. Since there are eight letters used, chance performance is 1/8 = 12.5%. In each case, classification performance was evaluated using a fivefold cross-validation procedure: for each of 10,000 iterations, we trained the classifier on 80% of the 192 stimuli, and tested it on the held out 20%. Decoding accuracy was calculated for each held-out 20% split as the percent of stimuli that were decoded correctly by the classifier. The mean and SD of decoding accuracy are shown in the *inset* of Fig. 2*D*.

### Model Fits for Responses to Longer Strings

Each neuron was tested with 10 strings at 4 distortions for each *n*-gram length (*n* = 2, 3, 4, 5, and 6). We used the firing rate of each neuron calculated as before to the 40 *n*-grams of a given *n*-gram length to fit two possible models based on single letters. For each *n*-gram (e.g., “yadgsT”), we compiled the corresponding predicted single letter responses from the multiplicative model (shape × distortion × location) (e.g., to “y” at location 1, “a” at location 2, etc.). Using the raw firing rates to single letters yielded qualitatively similar results.

For the additive model, the response to each *n*-gram was given by a linear sum of the responses to single letter. To fit the additive model, we compiled the *n*-gram responses (for each length) and the corresponding single letter responses into the matrices **y** and **X**, respectively, where **y** is a 40 × 1 vector containing the responses to the 40 *n*-grams of that length in odd reps, **X** is a 40 × *n* matrix containing the corresponding single letter responses at each of *n* retinal locations together with a constant term. We then modeled the full set of 200 unique *n*-gram responses as a single equation **z = Ab**, where **z** is a concatenation of all **y** *n*-gram response vectors at each length (*n* = 2, 3, 4, 5, and 6) and the letter response matrix **A** was a 200 × 25 matrix in which corresponding single letter responses at each length were entered into a separate set of columns. In other words, the matrix **A** had a total of 25 columns made up of 3 columns for bigram letter responses (2 weights and 1 constant), 4 columns for trigrams (3 weights and 1 constant), and so on. The regression weight vector **b** is a vector of 25 unknowns representing the weights of each location within the *n*-gram for each particular *n*-gram length.

For the multiplicative model, the response to each *n*-gram was given by a generalized product of the response to single letters. For example, the response to the bigram ab is given by $r_{ab}=\;w3\times r_{a}^{w1}\times r_{b}^{w2}$, where *r_a_*, *r_b_* are the responses to the single letters, and *w*1, *w*2, and *w*3 are free parameters. To fit the multiplicative model, we note that taking the logarithm on both sides yields a linear model—the bigram equation is equivalent to log(r*_ab_*) = log(w3) + w1 × log(r*_a_*) + w2 × log( r*_b_*), which is a linear equation. We, therefore, solved a linear regression of the form **z = Ab,** where z = log(y) and A = log(X), where **y** and **X** are the vector and matrix defined earlier for the additive model, and **b** is a vector of unknowns. This was done for each *n*-gram length separately. We then calculated the predictions of the multiplicative model by taking the inverse logarithm of the predictions from this model, and combined them across *n*-grams to yield a composite predictions across all *n*-grams.

**Figure 3.**
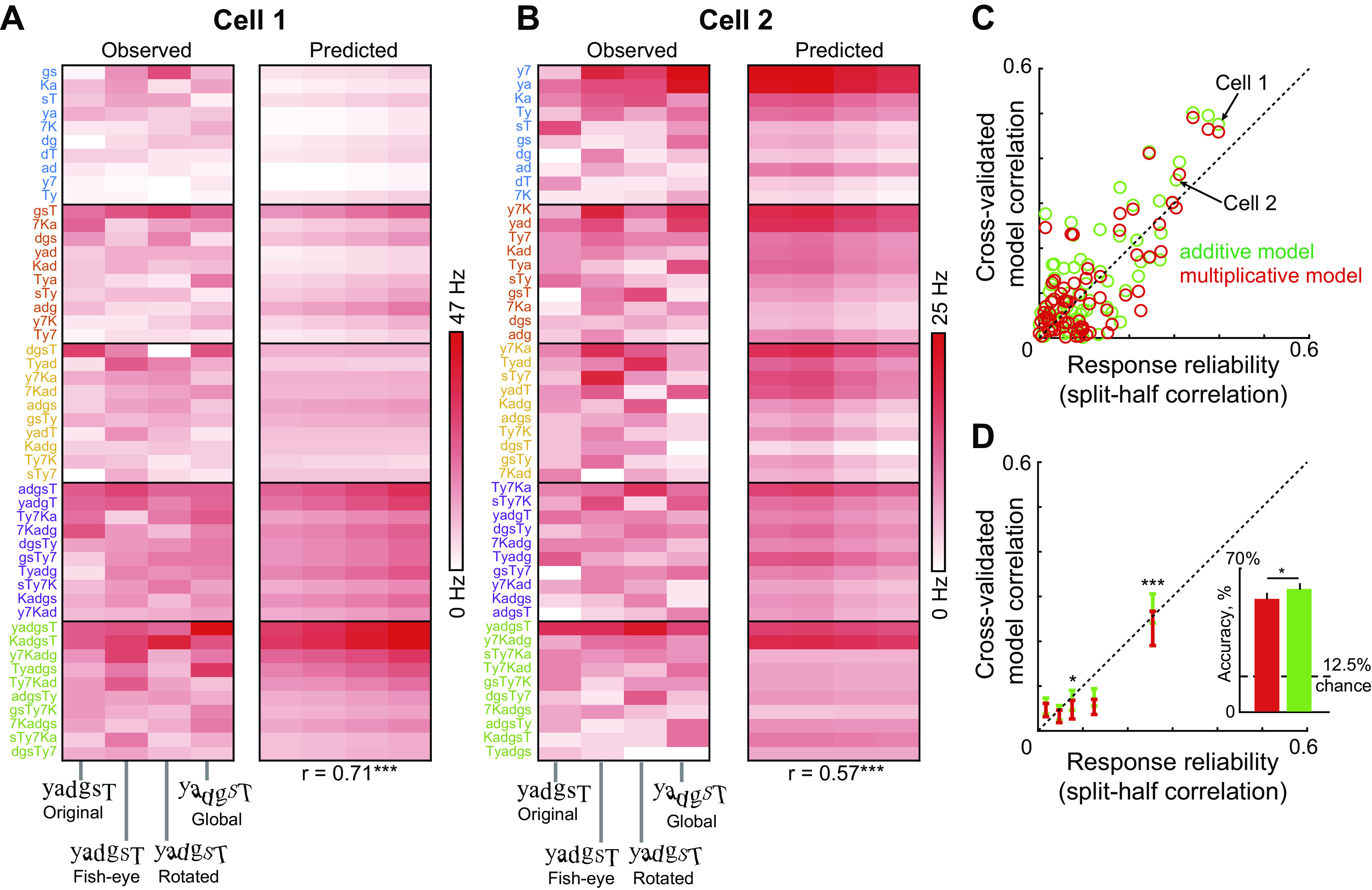
Responses to *n*-grams predicted from single letters. *A*, *left*: colormap of observed responses of Cell 1 (averaged across all trials) to all *n*-grams (arranged along the *y*-axis from best to worst) across all distortions (along the *x*-axis). *Right*: predicted response from the compositional model trained on all trials in which the response to each *n*-gram is modeled as a weighted sum of the single character response at that location (correlation with observed response: *r* = 0.71, *P* < 0.05, depicted below). *B*: same as A but for Cell 2. Note that *n*-grams are resorted along the *y*-axis from best to worst for this cell. *C*: cross-validated model fit for the additive (*green*) and multiplicative (*red*) models for neural responses to *n*-grams plotted against response reliability (i.e., split-half correlation) across neurons with positive reliability. In the multiplicative (additive) model, responses to multiple letters is predicted as a product (sum) of single letter responses. *D*: progression of model correlation with response reliability for neurons at each 20th percentile of response variability. Error bars represent means ± SE in each bin. Asterisk indicates statistical significance for additive vs. multiplicative model comparisons in each group (*P < 0.05, ***P < 0.0005). *Inset:* to investigate whether the slight advantage for additive separability of string responses at the single cell level has any functional advantage at the population level, we trained linear classifiers to decode letter identity using the multiplicative and additive model predictions separately (see methods). Decoding accuracy is shown for the multiplicative population (*red*) and additive population (*green*). Error bars represent standard deviation of accuracy. Asterisks represent statistical significance calculated as the proportion of the 10,000 cross-validated splits in which the additive population accuracy was lower than the multiplicative population (*P < 0.05).

To avoid overfitting, we trained each model on the average firing rate on odd-numbered repetitions and calculated its correlation on the average firing rate on the even-numbered repetitions, and vice-versa. We averaged the two correlations from the odd- and even-numbered repetitions to obtained a single cross-validated model fit. We compared this model fit against the split-half correlation for each neuron in Fig. 3*C*. For Fig. 3*C*, we excluded 50 neurons with high response variability that caused them to have a negative split-half correlation.

### Letter Decoding Using *n*-gram Responses in Multiplicative and Additive Populations

For 89 visual neurons with non-negative response reliability, we fit additive and multiplicative models as described in METHODS for each neuron using its average firing rate (across all trials) and created two 89 × 200 response matrices (89 neurons × 200 *n*-gram stimuli) for each population. We also created corresponding 200 × 1 label vectors corresponding to the letter shown at each position. We then trained 10,000 iterations of fivefold cross-validated linear letter decoders as described in the section immediately below. The means and standard deviation of letter decoding accuracy using *n*-gram responses in multiplicative and additive populations is shown in the *inset* of Fig. 3*D*.

### Decoding Character Identity for Every Location in an *n*-Gram

For each neural population, we trained linear classifiers to decode the individual letters at each retinal location. To simultaneously decode all *n*-grams, we included a blank as a character so that there were nine possible labels (8 letters + 1 blank) at each retinal location. For every retinal location, an *n*-gram response was assigned the label corresponding to the character contained at that location, i.e., the *n*-gram “adgsT” would have the letter labels (“blank,” “a,” “d,” “g,” “s,” and “T”), respectively, in retinal locations one through six. The number of labeled training instances for each label class were balanced to avoid classifier bias. In this manner, we trained six nine-way linear classifiers for each of the six retinal locations. Decoding accuracy is reported throughout as the average performance across these six retinal locations. Since there are nine classes in the classifier, chance performance is 1/9 = 11%. Since blank spaces occurred more frequently at eccentric retinal locations, we calculated the average number of observations across the eight-character classes at a given retinal location, and sampled an equal number randomly from the set of responses corresponding to blank spaces. We did this to prevent the classifier from learning trivial decision boundaries such as classifying all test observations as blanks.

Since the number of feature dimensions could exceed the number of observations, such as when we balanced letter and blank classes, we performed a principal component analysis (PCA) on the firing rate matrices to retain dimensions that captured 95% variance in the data. We then trained linear classifiers for letter classification at each position, and performance was evaluated using a fivefold cross-validation procedure. We trained each classifier on 80% of the observations and tested it on the held out 20%, and repeated it five times. Decoding accuracy was calculated for each held out 20% split, and the means and standard deviation of the accuracy across splits is shown in the figures (Fig. 3*D*, Fig. 6*B*, and Fig. 7*C*).

### Creation of Artificial Neurons That Had Emergent Information

We created populations of artificial neurons having one of three different possibilities of emergent information (Fig. 5). In the first type, each neuron in the pool had a conjunction response for one of the ten possible bigrams and no response to single letters. The responses were chosen to follow a Poisson process with a mean so that its response reliability matched that of one unique neuron in our set of 139 visually responsive neurons. The second type of neurons also had conjunction coding for one of ten possible bigrams, but had baseline Poisson firing for all other letters and strings that did not contain that bigram. The baseline firing was set to 30% of the mean firing rate of the corresponding biological neuron. In this case as well, response reliability of each neuron in the synthetic neuron pool was matched to one of the real biological neurons. Finally, we created neurons of a third type that responded to longer strings either as the sum of responses to constituent letters, or if the longer string contains the special bigram, as the sum of responses to the bigram and the other letters, with divisive normalization. Letter and bigram responses were scaled so that overall response reliability of each artificial neuron matched to each unique biological neuron.

### Creation of Artificial Neurons with Separable Responses

We created a population of 1,000 artificial neurons with separable responses as follows (Fig. 6*A*). Each neuron was initialized with tuning for letter, location, and distortion that could be sharp or broad. For broad tuning, we selected responses to be uniformly sampled from 0 to 1 generator (rand function in MATLAB). For sharp tuning, we chose only one response to equal 1 and the remaining to be 0 according to a sparse matrix generator [sprand function in MATLAB, with density 1/(*n*-gram length)]. For mixed tuning, we created neurons that were selective from only one or more letters but not to the remaining ones (sprand function in MATLAB, with density 1/*k*, *k* = 1, 2, …, 6). A given neuron was chosen as sharply (or broadly) tuned for all three attributes (shape, location, and distortion) since IT neurons are known to have this property (21). For the mixed population of neurons, each neuron was randomly assigned (with equal probability) to be sharply or broadly tuned. We then calculated the response of each neuron to distorted letters and strings by applying either additive or multiplicative. Finally, the response to *n*-grams was calculated as the sum of the responses to single letters at the corresponding retinal locations and divided by the number of letters (if divisive normalization was selected). In this manner, we created several groups of artificial neurons: broad tuning with multiplicative/additive integration with/without normalization, sharp tuning with multiplicative/additive integration with/without normalization, and mixed tuning with multiplicative/additive integration with/without normalization.

To truly test the capabilities of CAPTCHA decoding for each synthetic neuron population, we calculated the response of each group to 17,920 *n*-gram stimuli. We created this multiletter *n*-gram dataset from the same eight characters we used in our original set of stimuli. For *n*-gram lengths varying from two to six, we first assigned characters without replacement to the same adjacent retinal locations that were used in our original set of *n*-grams (e.g., central-most two locations for bigrams), nonletter positions were filled with blanks. This procedure yielded 8 × 8 = 64 unique strings for bigrams (e.g., **aa**, **ab**, where * denotes blanks). In this manner, we obtained 64, 512, 4,096, 32,768, and 262,144 strings of lengths two to six, respectively. We then performed circular shifts on these strings to obtain strings with new spatial mappings for adjacent characters, for example, the string **ab** gave the unique strings ***ab*, ****ab, b****a, and ab**** using this procedure. We then created more unique strings by letters across retinal locations obtained more strings, such as *a**b*, *a***b, and included them in the dataset if they were not already present. At this stage, we obtained 28,736, 3,584, 4,416, 32,768, and 262,144 strings of lengths two to six, respectively. To avoid training biases, we sampled from the longer string sets to obtain a dataset with 17,920 unique strings with 3,584 *n*-grams of every length.

### Separability in Deep Convolutional Neural Networks

We analyzed four deep convolutional neural networks for their responses to our stimulus set (Fig. 7). The first network served as a baseline and is a VGG-16 architecture trained for object classification on the ImageNet dataset (22). The VGG-16 model we used had five convolution stages that had 3 × 3 filters and 64, 128, 256, 512, and 512 channels, respectively, and each convolution was accompanied by 2 × 2 max-pooling and RelU (Rectified linear unit) operations (23).

We also selected three deep networks trained for text decoding using images as inputs (24, 25). These networks were trained in these studies using a large-scale dataset created by generating words of lengths up to 23 characters and rendering them using a variety of distortions, fonts, border/shadow rendering, noise, and background information statistics. These three models have a common backbone architecture consisting of input (32 × 100 × 1), convolutional (32 × 100 × 64, 16 × 50 × 128, 8 × 25 × 256, 4 × 13 × 512), and fully connected (1 × 1 × 4,096) stages, before being connected to either 23 output stages (each 1 × 1 × 37) for *charnet*, a single *n*-gram output stage (1 × 1 × 10,000) for *ngramnet*, or a single word output stage (1 × 1 × 90,000) for *dictnet*.

The first network, which we denote as *charnet*, was trained to correctly identity the letter or number at each retinal location in words of up to length 23. Words were left aligned in a large-scale training data set of ∼8 million words. The final decision layer for *charnet* comprised of 851 nodes corresponding to character identities (26 letters + 10 numbers + 1 blank space) for each of 23 locations. The second network, which we denote as *ngramnet*, was trained to detect the presence of every *n*-gram present in a word out of a possible 10,000 *n*-grams. For example, when the input image contains the string “ad,” this network will return the *n*-grams (“a,” “d,” “ad”). The third network, which we denote as *dictnet*, was trained to identify whole words from a dictionary of 90,000 possible words.

Because information in all four networks passes through a fully connected stage with 4,096 nodes before the final classification stage, we selected this layer for our analyses. To analyze the separability of distorted letter strings in each network, we generated its activations for the stimuli in our neural recordings, and performed the exact same analysis as for real neurons.

## RESULTS

We recorded from 139 single neurons in the IT cortex of two monkeys performing a fixation task. We tested each neuron with a fixed set of stimuli which comprised CAPTCHA-like strings that were 2–6 letters long, each of which was presented at four distortions (see methods; Supplemental Fig. S1). The stimuli also included single letters presented at each retinal location and at each distortion.

We define a set of neural responses to be separable if they can be predicted as a simple function of independent components (Fig. 1). For example, the neural response to a particular shape at a specific retinal location is separable if it can be expressed as a function of a fixed underlying shape preference (independent of location) and a fixed underlying location preference (independent of shape). The function can be additive or multiplicative, or of course a more complex combination. By contrast, the neural response would be inseparable if the neuron responded to one shape at one location and another shape at another location, making it impossible to write down as a function of shape-only and location-only tuning. Here, we sought to distinguish between the simplest two forms of separability, namely, whether the function is a simple sum or product of the underlying components.

We asked two fundamental questions regarding the separability of neural responses to single letters and to multiple letters (Fig. 1). First, are neural responses to distorted letters and distorted strings separable into a sum or product of their tuning for shape and tuning for distortions (Fig. 1*A*)? Second, can neural responses to strings be explained as a sum or product of their responses to single letters (Fig. 1*B*)?

### Do IT Neurons Show Separable Responses to Distorted Letters and Strings?

We first characterized neural responses to single letters across retinal locations and distortions. The responses of two example IT neurons to letters at every retinal location and distortion are shown in Fig. 2, *A* and *B* (*left*). It can be seen that both neurons have roughly similar responses across rows and columns, suggesting that they preserve their selectivity for shape/position/distortion. To quantify this pattern, we fit the average firing rate of each neuron (across all trials) to a model where the response is a product of shape, location, and distortion tuning (see methods). The predicted responses for both neurons were significantly correlated with the observed response (Fig. 2, *A* and *B*, *right*). The observed and predicted responses for several other neurons is shown in Supplemental Fig. S4.

This pattern was true across the recorded population of neurons. We compared two models in which the predicted response is either a sum or product of shape, location, and distortion tuning. To avoid overfitting, we calculated a cross-validated model correlation for each neuron based on training the model on only half the trials and predicting responses in the held out half (see methods). This was done once for even and once for odd trials, for every neuron and the correlations between model predictions and held out trials were averaged to get the separable model performance for each neuron. This form of cross-validation ensured that our models did not see the data being predicted. We compared these cross-validated model predictions with the response reliability of each neuron, calculated as the split-half correlation, i.e., the correlation between the firing rates on odd- and even-numbered trials. The resulting plot of the cross-validated model correlation against response reliability is shown in Fig. 2*C*.

If overall CAPTCHA decoding by the neural population is driven by small numbers of neurons with highly consistent but inseparable responses, this would result in systematically worse model fits for neurons with high response consistency. However, we observed no such trend (Fig. 2*C*). Model correlations hewed close to the response reliability throughout, indicating that both models explained nearly all the explainable variance in the responses.

We note that in many cases, model correlations were higher than the response reliability, presumably because the model provides a compact description of the data (# parameters = 8 for letters, 6 for position, 4 for distortion = 18 parameters, which explain 192 responses) which effectively denoises the data. To confirm that this is indeed the case, we created a population of artificial neurons with multiplicative encoding of shape/location/distortions and with Poisson firing matched in reliability to the observed IT neurons, and repeated the model fits as before. The resulting model fits were systematically larger than the observed response reliability (average difference between model correlation and response reliability: 0.07 ± 0.1 across 139 model neurons, *P* < 0.0001 on a sign-rank test comparing these to a zero median). Finally, to confirm that the above results were not trivially due to sparse tuning for one or two outliers, we repeated the above analyses after excluding the best or the best two letters for each neuron, and obtained qualitatively similar results.

To compare the additive and multiplicative model performance systematically, we sorted neurons into equal-sized groups based on their response reliability, and compared the two models. Both models were comparable in their performance at every range of response reliability (Fig. 2*D*). However, for the most reliable group of neurons, the multiplicative model had a slight but significant advantage (Fig. 2*D*; model correlations, means ± SD: *r* = 0.48 ± 0.13 for multiplicative model, *r* = 0.47 ± 0.13 for the additive model, *P* < 0.05, sign-rank test across 19 neurons). Although this is a very small difference, this is to be expected since the sum and product of two numbers are always highly correlated.

### Does Multiplicative Separability Lead to Better Population Decoding?

Would the slight advantage for the multiplicative model, although statistically significant, offer any meaningful functional advantage? To investigate this possibility, we took the predictions of the additive and multiplicative models fit to each neuron, and trained linear classifiers on each population to decode letter identity (see methods). This revealed a large advantage for letter decoding in the multiplicative population (∼25% higher accuracy; Fig. 2*D*, *inset*). Thus a slight advantage in multiplicative separability at the single cell level leads to a large benefit in letter decoding at the population level. We obtained concordant results in our investigation using artificial neurons with multiplicative versus additive encoding below (Fig. 6).

### Can Neural Responses Be Explained by More Complex or Simpler Models?

Since the additive and multiplicative models themselves explain most of the explainable variance in the neural response, we did not consider more complex models. However, to explore this possibility further, we asked whether neural responses would be explained better by a combination of additive and multiplicative terms. To this end, we fitted the neural responses using a combination model where the response to a given shape/position/distortion is given by *r* = *w_1_r_a_ + w_2_r_m_ + w_3_r_a_r_m_*, where *r_a_* and *r_m_* are the additive and multiplicative model predictions, and *w_1_*, *w_2_*, and *w_3_* represent unknown weights. The resulting cross-validated model fits, evaluated in the same way as before, were not significantly different from the multiplicative model (model correlations, means ± SD: *r* = 0.06 ± 0.1, 0.11 ± 0.08, 0.18 ± 0.08, 0.22 ± 0.13, and 0.47 ± 0.13 for the combination model at the five levels of reliability shown in Fig. 2*D*; *r* = 0.06 ± 0.1, 0.11 ± 0.08, 0.18 ± 0.08, 0.22 ± 0.13, and 0.48 ± 0.13 for the multiplicative model; *P* = 0.37, 0.41, 0.07, 0.77, 0.14, sign-rank test comparing the full model vs. multiplicative model correlations for each group). Thus, neural responses are not explained better by more complex combinations of additive and multiplicative terms.

These results show that responses of IT neurons to distorted letters can be explained using a product of shape, location, and distortion tuning, but do not elucidate the relative importance of these factors. To investigate these issues, we repeated the above analyses by fitting simpler models that contained only shape, only location, or only distortion predictors, and to models including only contralateral or ipsilateral locations. This revealed that shape and location were the dominant contributors to the response, which is expected given that IT neurons are invariant to slight shape distortions. In addition, we observed that contralateral and ipsilateral positions contributed equally to the model fits, which is consistent with the fact that IT neurons generally have bilateral receptive fields (Supplemental Fig. S2).

Because we tested each neuron on longer strings at every possible distortion, we were also able to ask whether neural responses to *n*-grams across distortions was separable as the sum or product of *n*-gram and distortion tuning. Here too, we found that the multiplicative model outperformed the additive model for the more reliable subgroup of neurons (Supplemental Fig. S3). Finally, we confirmed that our results were qualitatively similar across neurons recorded from both monkeys (Supplemental Fig. S6).

### Do IT Neurons Show Separable Responses to Letter Combinations?

Next we set out to characterize neural responses to letter combinations. The responses of the same two cells to *n*-grams varying in length from two to six letters is shown in Fig. 3, *A* and *B*. It can be seen that Cell 1 responds to *n*-grams containing the letter “g” whereas Cell 2 responds to *n*-grams containing letters “7,” “d,” “T,” and “y”. These patterns suggest that responses to longer strings can be explained using single letter responses. To quantitatively assess this possibility, we asked whether the response of each neuron to *n*-grams of each given length can be explained as a location-weighted sum of multiplicatively reconstructed responses to single letters (see methods). The location-weighting accounts for the contribution of a given letter depending on its location within the full string. This model yielded excellent fits for the two example neurons (*r* = 0.71 for Cell 1, *r* = 0.57 for Cell 2, *P* < 0.0005; Fig. 3, *A* and *B*). The observed and predicted responses for several other neurons is shown in Supplemental Fig. S4.

We compared the predictions of this additive model with a multiplicative model, where the response to longer strings of each given length was a generalized product of reconstructed responses to single letters (see methods). Because the two models have the same number of free parameters, their fit to the data can be compared directly. As before, we plotted cross-validated model fits against the response reliability for each neuron. While both models showed better fits with increased response reliability, the additive model yielded better fits both across all neurons (model correlations, means ± SD: *r* = 0.11 ± 0.13 for the additive model; *r* = 0.08 ± 0.12 for the multiplicative model; *P* < 0.00005, sign-rank test across 89 neurons with positive response reliability) as well as for the most reliable group of neurons (model correlations, means ± SD: *r* = 0.23 ± 0.15 for the additive model; *r* = 0.20 ± 0.16 for the multiplicative model; *P* < 0.005, sign-rank test across 21 neurons). We obtained qualitatively similar results across neurons in both monkeys (Supplemental Fig. S6).

To investigate whether the slight advantage for additive encoding of multiple letters confers a large functional advantage for letter decoding, we proceeded as before to take the predictions of the additive and multiplicative model fits from each neuron, and trained linear classifiers on each population to decode letter identity (see methods). This revealed a small but significant advantage (∼5% increase in accuracy) for the additive population (Fig. 3*D*, *inset*). We obtained similar results for artificial neurons (see *Is Separability Sufficient for CAPTCHA Decoding*? below).

Next, we examined the estimated summation weights of the additive model at each retinal location. While there was considerable diversity across neurons, summation weights for the middle letters were smaller than for the extreme letters (Supplemental Fig. S6). Moreover, the relationship between n-gram responses and the sum of single letter responses followed a characteristic 1/*n* progression (Supplemental Fig. S6). This is consistent with the divisive normalization observed previously in IT neurons (16).

To be sure that model fits are not artificially high due to overfitting, we shuffled the *n*-gram responses so that the association between an *n*-gram and its constituent letters was no longer systematic, and repeated the additive model fit. This abolished the close correspondence between model fits and the response reliability (Supplemental Fig. S2).

### Do IT Neurons Show Systematic Errors Indicative of Conjunction Tuning?

**Figure 4.**
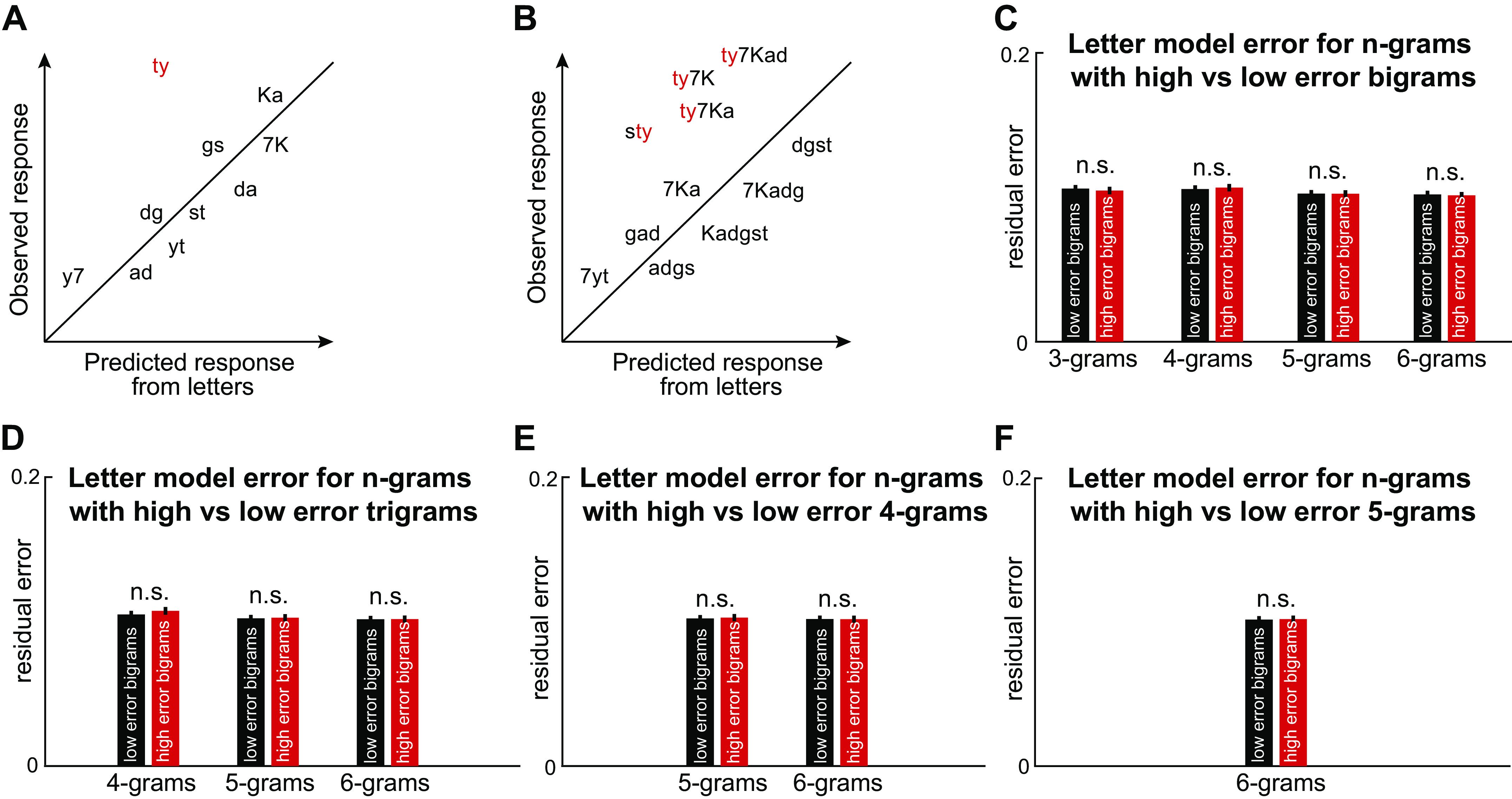
Error analysis to identify conjunction responses. *A*: consider a neuron selective for the bigram “ty”– by definition it would mean that its response to “ty” systematically deviates from the prediction from single letters. *B*: if this neuron is consistently detecting the bigram “ty” its response to any longer string containing “ty” should deviate in the same direction. *C*: to assess this possibility, we fit the compositional model to the observed response across all trials (after normalizing to the maximum firing rate) for each *n*-gram length and calculated the absolute difference between the observed and predicted response for each *n*-gram. Next, we sorted bigrams according to the bigram model error into two groups, one with higher model errors and the other with lower model errors. We then calculated the average model error (absolute value of observed minus predicted response after normalizing to the maximum firing rate) for longer strings containing high-error bigrams and low-error bigrams after fitting the linear model to strings of each length. Error bars represent means ± SE across neurons. Since we are comparing *n*-gram model error for longer strings sorted into two groups based on an independent criterion, this procedure requires no cross validation. We found no systematic difference in error at each *n*-gram length (*P* > 0.05, sign-rank test across neurons, marked as “n.s.”). *D*: same as *C* but for high- and low-error 3-grams. *E*: same as *C* but for high and low-error 4-grams. *F*: same as *C* but for high- and low-error 5-grams.

The above-mentioned analysis shows that neural responses to letter strings is well approximated by a sum of single letter responses, but neurons might still show systematic model errors indicative of selectivity for letter combinations. We reasoned that, if a neuron shows a deviant response to a particular bigram that is not predicted by its response to single letters (e.g., the bigram “ty” as shown in Fig. 4*A*), then its response to longer strings containing the same bigram (e.g., “sty,” “ty7K,” etc. as shown in Fig. 4*B*) will also not be predictable from single letters. If such conjunction responses were prevalent throughout the neural population, the model error for trigrams containing these high-error bigrams will be systematically larger than trigrams that do not contain such bigrams.

We tested this prediction by dividing each *n*-gram set into high and low-error *n*-grams, and compared the model error for longer strings containing these *n*-grams. Contrary to this prediction, we found no such systematic difference in model error (Fig. 4, *C*–*F*), suggesting that errors in longer *n*-grams are not systematically related to errors in shorter bigrams. This finding is consistent with the separable encoding of attributes described in the earlier sections.

### Can Deviations from Separability Be Detected Given Our Experimental Design?

The above-mentioned results show that IT neural responses to distorted letter strings are highly separable, but it could be that deviations from separability cannot be detected using our experimental design given the choice of *n-*grams used and the response reliability present in our neural data. To investigate this possibility further, we created several types of artificial neurons whose responses to *n*-grams are not predictable from single letters, but whose reliability is matched exactly to the recorded neural population. We note that there are an infinite number of ways of creating inseparable responses, and it is impossible to evaluate all of them. We, therefore, evaluated one specific form of inseparability, namely, conjunction coding, where neurons respond to specific letter combinations but not to the constituent letters.

**Figure 5.**
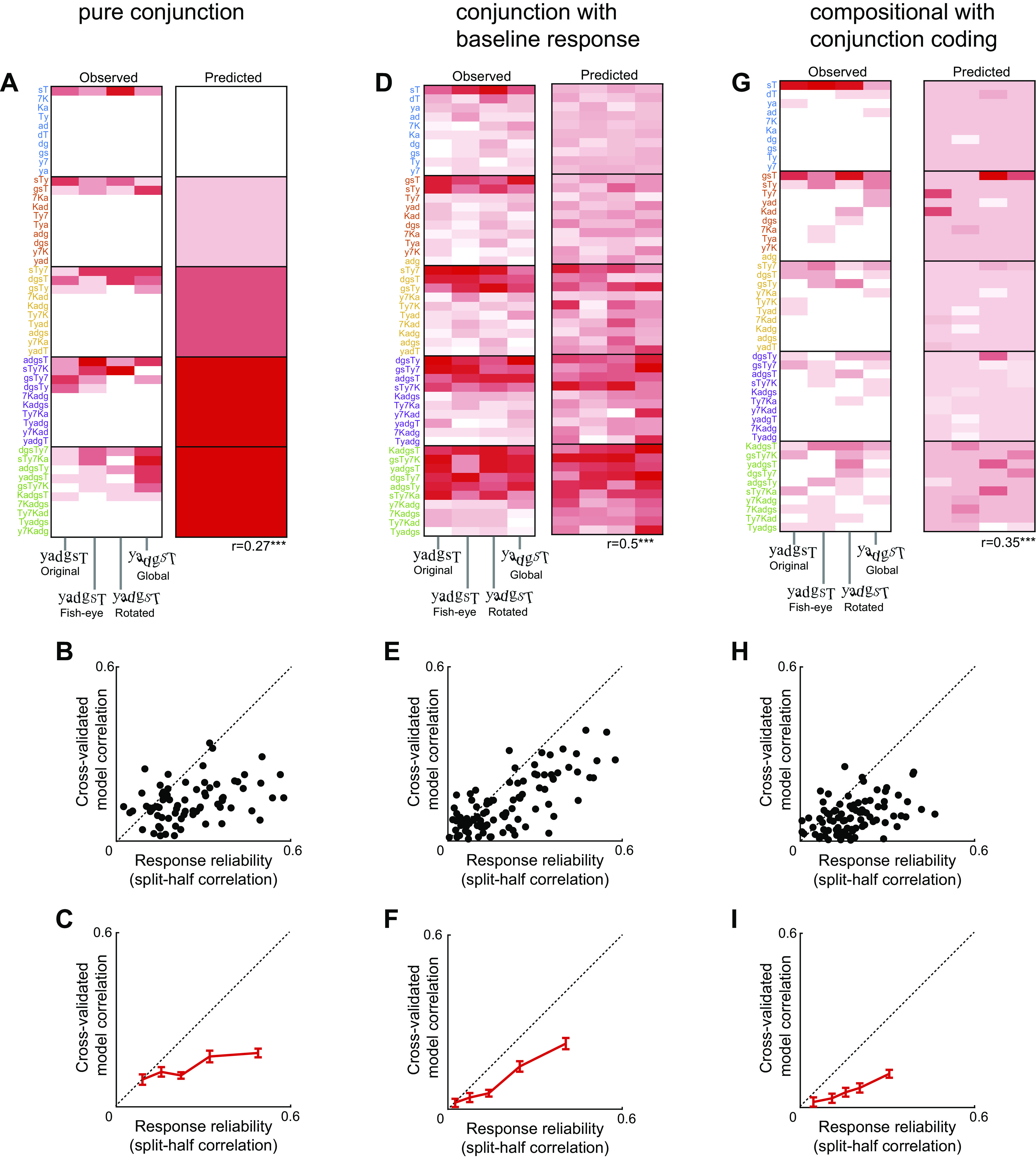
Detection of conjunction coding using our study design. *A*: here, we created artificial neurons with conjunction tuning for one bigram with response reliability matched to the real neurons. *Left*: the colormap shows one artificial neuron tuned to the bigram “sT” and any longer string containing this bigram, but with no responses to single letters. The responses of this neuron were chosen to follow a Poisson process with mean chosen so that its response reliability matched that of a real neuron. *Right*: predictions from a model in which *n*-gram responses are a sum of single-letter responses. Note that model predictions are constant for each *n*-gram length since single letters have no predictive power. ***P < 0.0005. *B*: cross-validated model predictions plotted against response reliability for these artificial bigram coding neurons. It can be seen that model correlation deviates from the response reliability throughout. *C*: progression of model correlation in *B* for each 20th percentile of response reliability. Error bars represent means ± SE in each bin. *D*: here, we created artificial neurons with conjunction coding to one bigram as before, but with baseline Poisson firing for all other letters and strings that do not contain this bigram (set to 30% of the mean firing rate of the corresponding real neuron). The colormaps show the observed (*left*) and predicted (*right*) responses of one such neuron. *E*: cross-validated model correlation plotted against the response reliability for these artificial neurons with conjunction coding for one bigram and baseline Poisson firing. *F*: progression of model correlation in *B* for each 20th percentile of response reliability. *G*: Here, we created artificial neurons with conjunction coding for a particular bigram but with compositional encoding of all longer strings. Specifically, each artificial neuron has the same letter tuning as its matched biological neuron, and responded to longer strings either as the sum of responses to constituent letters, or if the longer string contains the special bigram, as the sum of responses to the bigram and the other letters, with divisive normalization. The letter response and bigram response were scaled such that the overall response reliability of the artificial neuron was matched to one unique real biological neuron. *H*: cross-validated model correlation plotted against the response reliability for these artificial neurons with conjunction coding for one bigram and compositional encoding of all longer strings. *I*: progression of model correlation in *H* for each 20th percentile of response reliability.

We evaluated our ability to detect conjunction coding of three broad types of neurons given our experimental design and response reliability. In the first type, each neuron responds to a specific bigram but not to other *n*-grams and constituent letters (Fig. 5*A*). In the second type, each neuron responds only to the presence of a specific bigram but has a baseline response for other letters and *n*-grams (Fig. 5*D*). In the third type, each neuron responds to only one specific bigram but its response to longer strings is predictable from the bigram response and the other letter responses (Fig. 5*G*). For all three types of neurons, model predictions deviated consistently away from response reliability throughout (Fig. 5).

We further asked whether these artificial neurons with conjunction coding could be detected using our error analysis from Fig. 4, in which we compared model errors for *n*-grams containing high versus low error *n*-grams. We performed this comparison for the two types of conjunction neurons that had nonzero responses to letters. Here too, we found that 75% of the comparisons (15/20) yielded significant model error differences, confirming that this error analysis also can reliably detect inseparability in the neural response.

We conclude that at least one form of inseparability—namely, conjunction coding or coding for combinations of letters—can be detected using our experimental design and the observed response reliability in IT neurons.

### Is Separability Sufficient for CAPTCHA Decoding?

So far we have shown that IT neurons encode distorted letter strings as a product of shape and distortion tuning, and encode letter combinations as a sum of single letters. However, they do not establish that CAPTCHAs can be solved using these responses, or that they can be solved if responses are separable.

To address these issues, we first asked whether CAPTCHAs can be solved using the responses of IT neurons to the distorted letter strings. To this end, we trained a nine-way linear classifier to decode character identity at every retinal location (see methods). Since each retinal location could have one of eight possible letters or be a blank space, chance performance for this decoding scheme is 1/9 or 11%. We used fivefold cross-validation to avoid overfitting. As expected, decoding accuracy using the recorded population of neurons was well above chance (average accuracy across 6 retinal locations = 29%).

The earlier result shows that we can decode letter identities successfully using a small population of neurons, but it is not clear how this decoding accuracy would scale with a larger population of neurons. It is also not clear whether the decoding accuracy is being driven by neurons with separable or inseparable responses. To resolve this issue, we asked whether CAPTCHAs can be decoded by a population of synthetic neurons with separable encoding.

**Figure 6.**
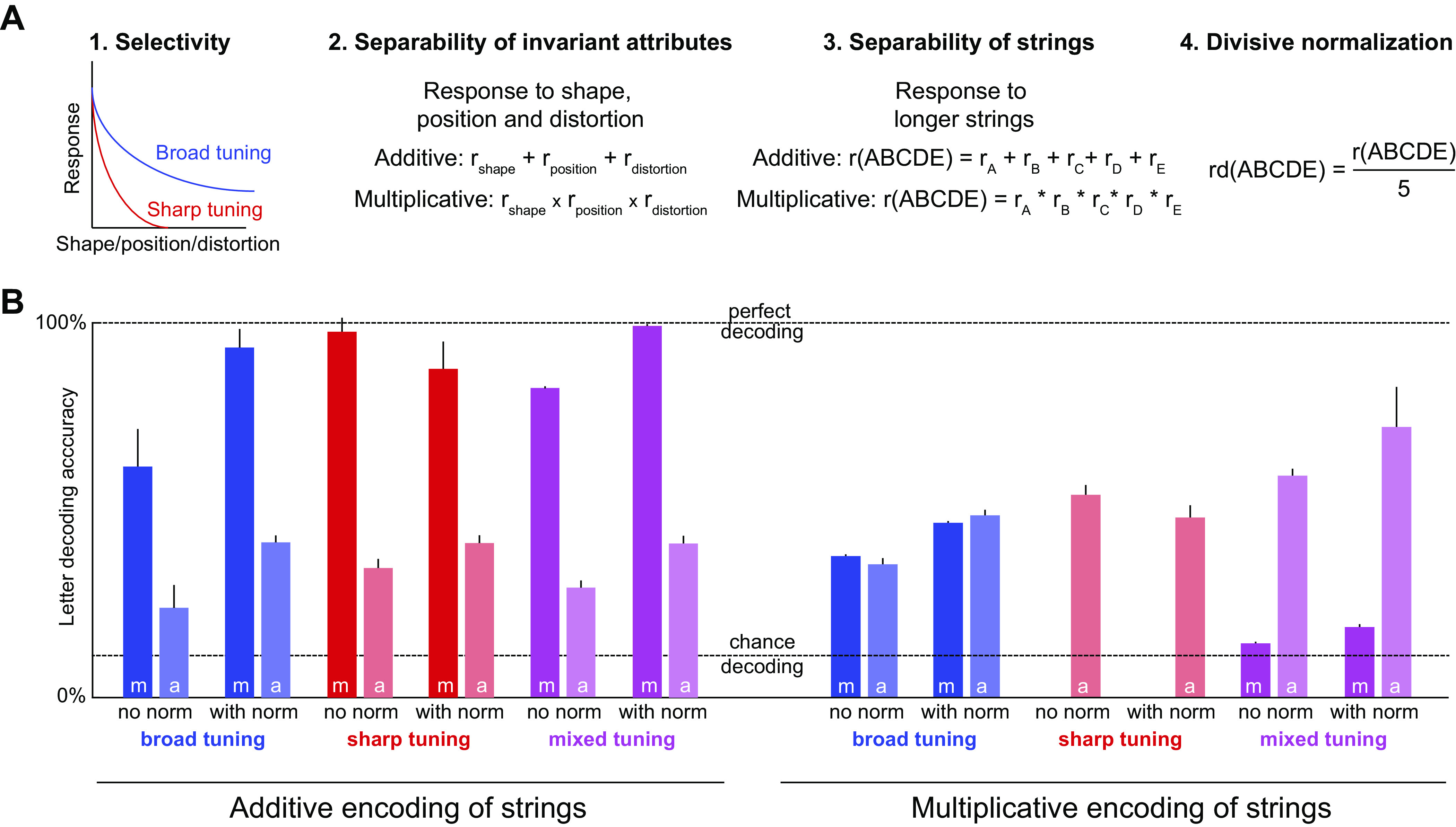
Response separability is sufficient for solving CAPTCHAs. *A*: creation of artificial neurons with varying choices of separability. *Step 1*: we created 1,000 artificial neurons with either sharp or broad tuning for letter, location, and distortion. *Step 2*: we calculated responses to distorted letters using either additive or multiplicative combination of shape, location, and distortion tuning. *Step 3*: we calculated responses to multiple letters as either a sum or product of single letter responses. *Step 4*: we either did or did not apply divisive normalization on responses to multiple letters. In this manner, we created separate neural populations containing every possible combination of these choices. *B*: we tested the CAPTCHA solving ability of each population of artificial neurons on a large set of 17,920 *n*-grams (see methods). To evaluate this neural population for CAPTCHA decoding, we trained a linear classifier to decode which of 9 possible characters (8 letters + 1 blank) were present at each retinal location in each *n*-gram. The resulting average character decoding accuracy is shown for each neural population created with a specific set of choices (*a* and *m* represent additive and multiplicative encoding of shape × position × distortion respectively; *norm* represents divisive normalization). Error bars represent standard deviation over 100 iterations of 1,000 randomly chosen neurons. It can be seen that multiplicative encoding of invariant attributes, together with additive encoding of strings, can produce perfect CAPTCHA decoding. The missing accuracy bars for highly sparse multiplicative models (sharp tuning with norm and no norm) are due to zero neural responses produced by multiplying their sharply tuned responses to various attributes. CAPTCHA, completely automated public Turing test to tell computers and humans apart.

We created 1,000 synthetic neurons that had either broad or sharp tuning for shape, location, and distortion (see methods; Fig. 6*A*). We calculated neural responses to shape-location-distortion combinations either using an additive or multiplicative function. Their responses to longer strings was taken simply as a sum of their responses to single letters, and finally we included divisive normalization whereby the net response to longer strings is divided by the string length. To provide a tough challenge of the CAPTCHA solving ability of this neural population, we created a large set of *n*-grams (*n* = 17,920) of varying string lengths, which could even contain spaces between letters (see methods). We then asked whether these separable neural responses could be used to decode character identity at each location as before (see methods). In this manner, we created several groups of neurons: sharp/broad/mixed tuning with multiplicative/additive integration with/without normalization (see methods).

The resulting character decoding accuracy for each of these artificial neuron populations is summarized in Fig. 6*B*. We observed several interesting patterns. First, decoding accuracy was always better when shape and distortion tuning were multiplicatively combined. This is because multiplying two signals enables efficient decoding compared to adding them, as we have reported previously (13). Second, decoding was better when responses of neurons with broad or mixed tuning were divisively normalized than when they were not. This is because normalization avoids excessively large responses to increasing content in the visual field. Since *n*-grams of different lengths can have the same character at a given location (e.g., “7yKa” and “Ty7Kad” both have “y” in the second location), longer *n*-grams will automatically have higher firing rates in the absence of divisive normalization and will result in two different read-outs for the same character label. Third, decoding is worse when neurons with sharp tuning are subjected to divisive normalization, which happens because sharply tuned neurons respond to only a few retinal locations and divisive normalization results in excessive scaling by the total string length. Finally, and most importantly, we obtained near-perfect CAPTCHA decoding accuracy with a mixed population of neurons with sharp and broad tuning.

We also asked whether additive encoding of multiple letters confers an advantage in letter decoding compared with encoding them multiplicatively. To this end, we repeated the same analyses as before, but using neurons in which string responses were a product rather than sum of letter responses. Here too we found a striking benefit for additive encoding of multiple letters (Fig. 6*B*).

We conclude that a particular type of response separability, namely, multiplicative encoding of invariant attributes and additive encoding of strings, is sufficient to solve CAPTCHAs perfectly.

### Do Deep Neural Networks Trained for Letter Recognition Show Separability?

So far we have shown that IT neurons show separable encoding of letter distortions and combinations. We then asked whether this is indeed an essential property of a visual system that aims to decode words and letters and is not merely restricted to biological neurons. Would separability be equally important even when the visual system was explicitly trained to identify entire words or *n*-grams as opposed to individual characters?

To address this issue, we analyzed the responses of four deep convolutional networks. The first network served as a baseline since it was trained on a standard object classification task and not for letter recognition (denoted as *imagenet*) (23). The remaining three of these were trained on a large-scale annotated data set of ∼8 million images generated from strings of variable lengths and different choices of fonts, projective transformations, and backgrounds (24). The networks were trained with three different goals: *1*) identifying the character at each of the 23 possible locations in the input word (denoted as *charnet*); *2*) enumerating all *n*-grams contained within the input word (denoted as *ngramnet*); and *3*) whole word identification from a dictionary of 90,000 words (denoted as *dictnet*). In all networks, we selected the penultimate fully connected layer (fc7, with 4,096 units) for further analysis, because this layer has the same dimensionality and is subsequently used by the decision layer. Our main goal in this analysis was to investigate whether separability emerged spontaneously in a family of popular deep neural networks trained for object and text classification. We chose variants of the VGG networks as they have been extremely popular for various visual categorization tasks including object, scene, face, and text classification. VGG based networks have an architecture that compares favorably with the stages and operations involved in feed-forward processing in visual processing (26, 27).

**Figure 7.**
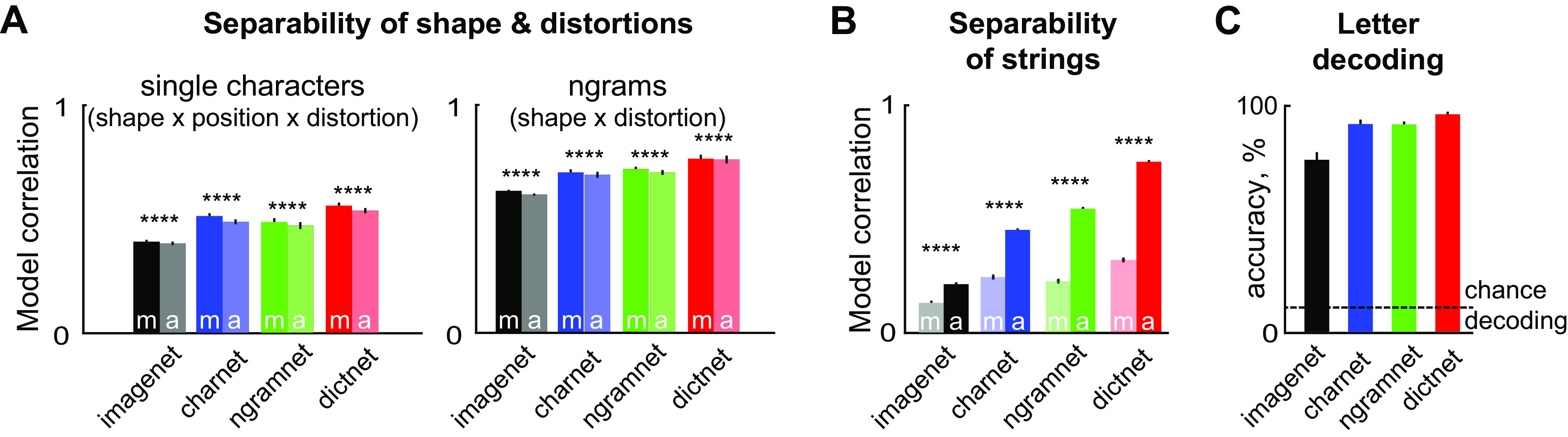
Compositionality in deep networks trained for text recognition. *A, left*: model correlations for additive (marked “a”) and multiplicative (marked “m”) models for distorted letters modeled as sum or product of shape, location, distortion tuning for each network. *Right*: model correlations for additive and multiplicative models for distorted strings modeled as sum or product of shape and distortion tuning for each neural network. Error bars represent means ± SE across neurons. Asterisks represent statistical significance calculated using a paired *t* test on the additive and multiplicative model correlations across all 4,096 units (****P < 0.00005). *B*: average model correlations for additive and multiplicative models for strings modeled as sum or product of single letter responses for each neural network. *C*: character decoding accuracy across the stimulus set used in the neural recording experiment for each neural network. Error bars represent means ± SE over character decoding accuracy over 6 retinal locations.

We next analyzed the activations of each network to the stimuli used in the neural recordings. As before, we fit additive and multiplicative models to predict the response of each unit in the fc7 layer to single letters across retinal locations and distortions. In all four networks, unit activations were better fit by the multiplicative model (Fig. 7*A*). Interestingly, the *imagenet* network yielded lower model fits compared to the networks trained on word recognition, whereas the *dictnet* model fits were the highest among all networks. We obtained similar results on analyzing *n*-gram responses across distortions (Fig. 7*A*).

Next, we asked whether unit activations to longer *n*-gram strings in our dataset, can be explained using a sum or product of single letter responses. Here too, we observed a clear progression, whereby the *imagenet* units yielded the least separability, and *dictnet* units were most separable (Fig. 7*B*). However, in all cases, model correlations are well below 1, suggesting that neural responses are not fully separable despite training. Such emergent compositionality has been observed in high-performing deep networks trained for natural scene categorization (28). Importantly, the responses of all networks to multiple letters was predicted better by the sum rather than product of the single letter responses (Fig. 7*B*), exactly as observed in the biological neurons (Fig. 3*D*).

To verify that these results were not artificially significant due to the large number of units in the deep network layers compared with real neurons, we repeated the above-mentioned analyses by repeatedly sampling 139 units (same as the IT neural population) of the 4,096 units and obtained qualitatively similar results for both types of separability (95% and 98% of 1,000 repeated samples showed a significance of *P* < 0.0001, sign-rank test between multiplicative vs. additive model comparisons for letters and *n-*grams, respectively).

Since the network trained on complete word recognition (*dictnet*) shows the greatest separability, we wondered whether its performance on letter recognition would also be the best across all networks. To investigate this issue, we compared the performance of each network on nine-way decoding of character decoding at each retinal location as before on the responses to our original *n*-gram set (50 unique *n*-grams × 4 distortions). As expected, the performance of *dictnet* was better than the other networks (decoding accuracy: 74%, 92%, 93%, and 95% for *imagenet*, *charnet*, *ngramnet*, and *dictnet*; Fig. 7*C*).

Thus, training deep neural networks for word recognition leads to separable neural representations for distortions and letter combinations, that are exactly analogous to the biological IT neurons. We speculate further that imposing separability as a constraint during learning can lead to better character decoding performance.

### Can IT Neurons Decode Both Easy and Hard CAPTCHAs?

**Figure 8.**
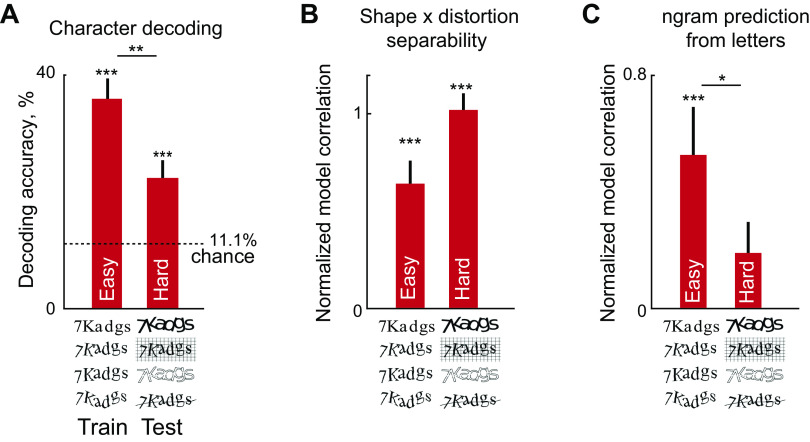
Decoding and separability for easy and hard CAPTCHAs by IT neurons. *A*: average decoding accuracy (of 9 characters across all retinal locations) for classifiers trained on the easy CAPTCHAs (*left*) and tested on the hard CAPTCHA strings (*right*). Error bars represent means ± SE across decoding accuracy across all *n*-grams. Asterisks above each bar represent the statistical significance of the decoding accuracy compared to chance decoding which is 1/9 = 11% (***P < 0.0005, based on calculating the binomial probability of exceeding this number of correct labels out of a total of 8 strings × 6 retinal locations × 4 distortions = 192 responses given a binomial distribution with *n* = 192, *P* = 1/9 = 11.1%). Asterisks straddling the two bars represent the statistical significance of the difference in decoding accuracy (**P < 0.005, rank-sum test for medians). *B*: normalized model correlation for the shape × distortion multiplicative model, for the easy and hard CAPTCHA strings. Error bars represent means ± SE across neurons. For each neuron, we calculated the correlation between observed responses on even-numbered repetitions and the model predictions based on odd-numbered repetitions, and divided by the split-half correlation to obtain a normalized measure. Asterisks above each bar indicate whether the median normalized correlation is different from zero (***P < 0.0005, based on a sign-rank test across 139 neurons). Although the hard CAPTCHA strings show greater separability, this difference was not statistically significant (*P* = 0.2, signed-rank test across 139 neurons). *C*: normalized model correlations for the *n*-gram responses predicted by single letter responses. Normalized correlations for hard CAPTCHA strings were not significantly different from zero (*P* = 0.09, signed-rank test), and this separability was significantly smaller than for the easy CAPTCHA strings (*P < 0.05, signed-rank test across 139 neurons). All other conventions are as in *B*. CAPTCHA, completely automated public Turing test to tell computers and humans apart; IT, inferior temporal.

The above-mentioned results were based on testing IT neurons with distorted letter strings with spatially separate letters and relatively simple distortions. However, we also included a small set of more challenging CAPTCHAs which contain overlapping letters or cluttered backgrounds (Fig. 8*A*). We predicted that letter decoding should also be possible in these hard CAPTCHAs, albeit at a weaker level.

To assess this possibility, we took linear classifiers to perform nine-way character decoding at each retinal location as before, and asked whether they could decode letter identity from the hard CAPTCHA strings. Decoding accuracy was weaker for the hard CAPTCHAs but still remained significantly above chance (Fig. 8*A*). Thus, CAPTCHAs that are hard for computer algorithms are also challenging for IT neurons.

Since hard CAPTCHAs are harder to decode, we wondered whether their responses are less separable with respect to distortions and string responses. To investigate this issue, we took the eight six-letter strings that were common to both the easy and hard CAPTCHAs (with 4 distortions each), and asked whether these responses were separable in terms of distortions and *n*-gram responses. We first fit a shape × distortion multiplicative model to the responses to the 8-grams across four distortions. To compare the model performance, we divided the model correlation of each cell by its split-half correlation for each set of stimuli. Both easy and hard CAPTCHAs were equally separable (Fig. 8*B*). Next, we asked whether the average response of each *n*-gram (on even-numbered repetitions) can be predicted using a weighted sum of the single letter responses (with weights learned from responses on odd-numbered repetitions), and normalized the model correlation as before. The resulting normalized model correlations are shown in Fig. 8*C*. As predicted, model correlations for hard CAPTCHAs were significantly smaller than for easy CAPTCHAs.

We conclude that hard CAPTCHAs are less separable than easy CAPTCHAs, which explains why they are harder to decode by the IT neural population.

## DISCUSSION

Here, we characterized the neural representation of distorted letters in IT neurons. We found two systematic encoding rules: neural responses to distorted letters was a product (but not sum) of distortion and shape tuning, whereas responses to letter combinations was a sum (but not product) of single letter responses. These two rules were sufficient to perfectly decode CAPTCHAs, and were present in neural networks trained for word recognition. In this section, we discuss these findings in relation to the literature.

Our main finding is that there is a systematic separable code for distorted letter strings in monkey IT neurons, consisting of two rules. The first rule was that neural responses to distorted letters is approximated well by a product (but not sum) of shape, position, and distortion tuning. This is consistent with recent observations that object identity and invariant attributes combine multiplicatively in IT neurons (13), and that IT neurons preserve their rank-order of shape preference across identity-preserving transformations (6, 9, 19, 20). The second rule is that neural responses to letter strings is a sum (but not product) of single letter responses. This is consistent with several previous studies showing linear summation in IT neurons for objects (16) and their parts (14, 15). However, our study extends these two rules to an important class of stimuli (letters) that undergo not only identity-preserving transformations such as size and position but also stylistic distortions such as seen in CAPTCHAs. These two rules together represent a double dissociation, which is noteworthy because there is no a priori reason for neurons to encode distortions multiplicatively and multiple letters additively or vice-versa. Indeed, we show that these two rules are the most efficient way to encode distorted letter strings and enable near-perfect CAPTCHA decoding. Finally, we also show that these two rules are present in deep neural networks trained for text recognition, which further confirms the utility of these two rules.

Why might it be efficient for IT neurons to encode information in this manner? A potential clue comes from the decoding results (Fig. 2*D* and Fig. 3*D*, *inset*)—letter decoding was better when invariant attributes (distortion and position) are combined with shape multiplicatively and when multiple letters were combined additively. To turn the argument around, we speculate that multiplicative separability might be driven by the need for invariant decoding while additive separability might be driven by the need for simultaneous decoding of multiple objects. This proposal is consistent with previous studies showing that preservation of rank-order preference is an important property for decoding both object identity and position across changes in size, position, and clutter (20).

Our findings are inconsistent with many previous studies suggesting that IT neurons are selective for specific combinations of features (9–11). However, these studies did not systematically compare object responses to their constituent parts or features. Doing this is nontrivial because isolating the parts from an object introduces new features where the object is cut—therefore, a different approach is needed whereby the part responses can be estimated indirectly from the whole object responses. When this has been done, whole object responses were indeed predictable from the constituent parts, both in IT neurons (14, 15) as well as in perception using visual search (15, 29, 30). We note however that there could be neurons with inseparable responses that carry other kinds of information, but the dominant trend in the population appears to be toward separability.

Our findings are also inconsistent with a recent study showing that *n*-gram responses are not entirely predictable from single letters in IT neurons (31). We speculate that the lower separability in that study could be due to different recording sites in IT (posterior/central IT in that study versus anterior/ventral IT in ours), different response noise distributions (multi-unit activity in that study vs. single units in ours), and different presentation times (100 ms in their study with no inter-stimulus interval vs. 200 ms on/off duration in ours), apart from of course stimulus and individual animal differences. These differences will need to be reconciled. However, we have gone beyond these specific results to show that separability in neural responses are sufficient to solve CAPTCHAs. This of course does not imply that inseparable neural responses, with neurons tuned for letter combinations, cannot solve CAPTCHAs or are not useful for other purposes; such codes might be required for further downstream processing, such as binding combinations of letters into syllables (2, 32). Rather, our results imply that, at least for the purposes of character decoding, response separability is sufficient. We speculate that there is a gradual progression in separability as well as invariance along the ventral pathway to enable efficient object recognition (5).

Our results have important implications for how exactly letter and word representations change with reading. First, we note that perfect CAPTCHA decoding using a perfectly separable neural population (Fig. 5), but encoding in IT neurons was not perfectly separable (e.g., Fig. 3*C*). Based on this we propose that reading could increase the overall separability of word representations. This was indeed observed recently in the anterior LOC (18), the homolog of IT cortex in humans. Whether this separability comes with exposure to single letters or to letter strings would be interesting to study. Second, we note that familiarity has widespread effects on IT neurons (33–35) and on human visual cortex (18, 36). We speculate that reading could lead to enhanced discrimination as letters become familiar, as has been observed recently (18). Third, reading is harder in the periphery than at the fovea, a phenomenon known as crowding (37, 38). We speculate that neural responses are less separable for stimuli in the periphery, or alternatively, this could be due to the well-known foveal bias of receptive fields in higher visual areas.

Our results also have important implications for machine vision. They indicate the specific representational rules that need to be embodied to solve CAPTCHAs. Indeed, there are deep network design and training principles that implement one or few of the following attributes, namely, compositionality, sparseness, and divisive normalization. An attempt has been made to bring in compositionality (39) into deep networks albeit maintaining a veridical mapping between sensory input and internal representation. Recent studies have shown recursive compositional architectures can also successfully decode CAPTCHAs (40). Randomly deleting connections during training iterations, also called drop-out, as well as nonlinearities such as rectified linear units (ReLUs) can bring in sparseness (41, 42). Finally, averaging CNN filter responses in a spatial neighborhood (mean-pool) is similar to divisive normalization for broadly tuned neurons and retaining only the highest response in a local neighborhood (max-pool) can approximate aggregation of highly sparse neurons (43). We propose that explicitly encoding separability could lead to vastly improved performance of deep neural networks on distorted letter recognition.

## DATA AVAILABILITY

All the codes and data required to replicate the results are publicly available on OSF at https://doi.org/10.17605/OSF.IO/2SGEU.

## SUPPLEMENTAL DATA

10.17605/OSF.IO/2SGEU Supplemental figures: https://doi.org/10.17605/OSF.IO/2SGEU.

## GRANTS

This work was supported by a Cognitive Science Research Initiative (CSRI) post-doctoral fellowship (to H.K.) from the Department of Science and Technology, Government of India and by Intermediate and Senior Fellowships (500027/Z/09/Z and IA/S/17/1/503081) from the DBT/Wellcome Trust India Alliance (to S.P.A.), and by the DBT-IISc partnership programme (to S.P.A.).

## DISCLOSURES

No conflicts of interest, financial or otherwise, are declared by the authors.

## AUTHOR CONTRIBUTIONS

H.K. and S.A. conceived and designed research; performed experiments; analyzed data; interpreted results of experiments; prepared figures; drafted manuscript; edited and revised manuscript; approved final version of manuscript.

## ENDNOTE

At the request of the authors, readers are herein alerted to the fact that additional materials related to this manuscript may be found at https://doi.org/10.17605/OSF.IO/2SGEU. These materials are not a part of this manuscript and have not undergone peer review by the American Physiological Society (APS). APS and the journal editors take no responsibility for these materials, for the website address, or for any links to or from it.
